# Semantic Segmentation Using Pixel-Wise Adaptive Label Smoothing via Self-Knowledge Distillation for Limited Labeling Data

**DOI:** 10.3390/s22072623

**Published:** 2022-03-29

**Authors:** Sangyong Park, Jaeseon Kim, Yong Seok Heo

**Affiliations:** 1 Department of Electrical and Computer Engineering, Ajou University, Suwon 16449, Korea; mailhoho@ajou.ac.kr (S.P.); jskim159@ajou.ac.kr (J.K.); 2 Department of Artificial Intelligence, Ajou University, Suwon 16449, Korea

**Keywords:** semantic segmentation, limited training data, self-knowledge distillation, regularization

## Abstract

To achieve high performance, most deep convolutional neural networks (DCNNs) require a significant amount of training data with ground truth labels. However, creating ground-truth labels for semantic segmentation requires more time, human effort, and cost compared with other tasks such as classification and object detection, because the ground-truth label of every pixel in an image is required. Hence, it is practically demanding to train DCNNs using a limited amount of training data for semantic segmentation. Generally, training DCNNs using a limited amount of data is problematic as it easily results in a decrease in the accuracy of the networks because of overfitting to the training data. Here, we propose a new regularization method called pixel-wise adaptive label smoothing (PALS) via self-knowledge distillation to stably train semantic segmentation networks in a practical situation, in which only a limited amount of training data is available. To mitigate the problem caused by limited training data, our method fully utilizes the internal statistics of pixels within an input image. Consequently, the proposed method generates a pixel-wise aggregated probability distribution using a similarity matrix that encodes the affinities between all pairs of pixels. To further increase the accuracy, we add one-hot encoded distributions with ground-truth labels to these aggregated distributions, and obtain our final soft labels. We demonstrate the effectiveness of our method for the Cityscapes dataset and the Pascal VOC2012 dataset using limited amounts of training data, such as 10%, 30%, 50%, and 100%. Based on various quantitative and qualitative comparisons, our method demonstrates more accurate results compared with previous methods. Specifically, for the Cityscapes test set, our method achieved mIoU improvements of 0.076%, 1.848%, 1.137%, and 1.063% for 10%, 30%, 50%, and 100% training data, respectively, compared with the method of the cross-entropy loss using one-hot encoding with ground truth labels.

## 1. Introduction

The goal of semantic segmentation is to predict the predefined class (or label) of each pixel, which is fundamental yet challenging in computer vision. Owing to its increasing importance, it is widely adopted in various applications using vision sensors, such as autonomous driving [1,2], 3D reconstruction [3], and medical image analysis [4,5]. In recent years, deep convolutional neural networks (DCNNs) have achieved significant performance improvements and have been the dominant solution for semantic segmentation. Since the introduction of FCNs [6], various architectures have been proposed, including U-Net [4], DeepLab [7,8,9,10], and PSPNet [11].

To achieve high performance, supervised learning in addition to a significant amount of training data are typically used in DCNN-based methods. Creating ground-truth labels for semantic segmentation requires more time, human effort, and cost compared with other tasks such as classification and object detection, because the ground-truth label of every pixel is required. Hence, it is practically demanding to train DCNNs using a limited amount of training data for semantic segmentation.

Generally, training DCNNs using a limited amount of data is problematic because it easily results in a decrease in the accuracy of the networks because of overfitting to the training data [12]. Overfitted models generate good results for the training dataset but subpar results for validation and test datasets, which are not used in training. However, many studies regarding semantic segmentation have focused mainly on improving the accuracy by assuming a significant amount of training data, whereas the problem of insufficient data for training has rarely been prioritized.

To mitigate the overfitting problem, the regularization method is widely used. This method includes early stopping [13], $L_{1}/L_{2}$-regularization [14], batch normalization [15], dropout [16], data augmentation [17,18,19] and regularizing the predictive distribution of DCNNs [20,21,22,23,24]. Specifically, regularizing the predictive distribution is an approach that regularizes the probabilities of network results. In this regard, various methods exist, such as label smoothing (LS) [20,21], confidence penalty (CP) [22,23], and knowledge distillation (KD) [25,26].

LS [20,21] generates a smoothed probability vector by adding a one-hot encoding vector using the ground truth and a uniform vector. It enforces the feature from the penultimate layer to be closest to the template of the correct class, while maintaining the same distance as those of the incorrect classes [20]. Hence, the probability generated using LS does not include the correlation information between classes. CP [22] increases the entropy of the prediction probability distribution by subtracting the entropy of the probability from the loss function. It does not include correlations between classes. In addition, it is problematic to further increase the entropy when the entropy of the probability distribution is already large, because this can render the label decision of the pixel ambiguous. KD improves the performance of the student network using the knowledge of the teacher network. However, a good teacher network is typically required to train the student network. Although the methods described above demonstrate good performances, they do not consider the problem of limited training data, and most of them are designed for classification problems, not semantic segmentation.

In this paper, we propose a new regularization method called a pixel-wise adaptive label smoothing (PALS) via self-knowledge distillation to stably train semantic segmentation networks in a practical situation, in which only a limited amount of training data are available. In this regard, we assume that the estimated probability distribution of each pixel exhibits certain relationships and correlations between all pairs of classes [27]. For example, the probabilities of bus and train classes exhibit higher correlations and closer relationships compared with those of bus and sky. Another intuition is that several pixels of the same class exist in an image. Hence, incorrect pixels can benefit from the correct pixels in an image by enforcing consistent distributions between pixels in the same class.

**Figure 1:**
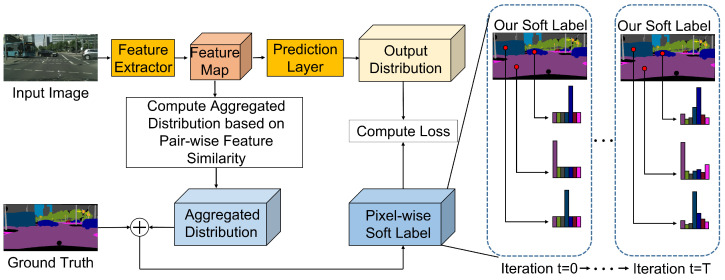
A schematic flowchart of our method. Our method aggregates distributions based on pair-wise feature similarity and generates a pixel-wise soft label by weighted sum of a one-hot encoding with ground truth label and the aggregated distribution for each pixel according to training iteration.

Based on these assumptions, the proposed method generates a pixel-wise adaptive soft label to regularize the estimated probability distribution of each pixel by fully utilizing the internal statistics of the pixels within an input image. Figure 1 shows a schematic flowchart of our method. In this regard, we compute a similarity matrix that encodes the affinities between all pairs of pixels. Based on this matrix, an aggregated probability distribution is computed by adaptively combining the probability distributions of correctly estimated pixels at other positions in an image. Our method compensates for insufficient data using soft labels obtained by aggregating the probabilities of other pixels in an image. However, in the early training step, the correctly predicted pixels are insufficient. Hence, we adaptively add a uniform distribution to the aggregated distribution as a function of the number of training iterations. As such, in the early step, a uniform probability has more weight than an aggregated probability. As training progresses, the aggregated distribution yields a larger weight. Although the aggregated distributions facilitate the reduction in the variance error of the estimation, they can result in increase in the bias error [28]. To reduce both bias and variance errors, we added one-hot encoded distributions with ground-truth labels to these aggregated distributions, which yielded our final soft labels.

**Figure 2:**
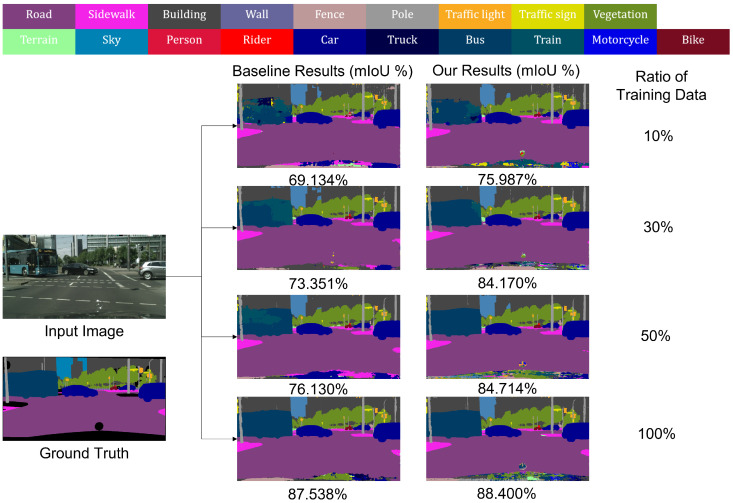
Comparative results of methods trained using various ratios of limited training data. Results of various ratios of training data including 10%, 30%, 50%, and 100% are shown. Value below each result represents mIoU.

Figure 2 shows the results of our proposed method and the conventional cross entropy (CE) method [10] for various ratios of limited training data on the Cityscapes dataset [29]. We used the same network as DeepLab-V3+ [10], hyperparameters, and a limited training data to compare those methods. The CE method, which involves less training data, predicts well for load, sidewalk, car, and vegetation classes, but not for bus classes. This is because the pixels for the bus class are fewer in all the training data, and the number of bus class pixels is further reduced in the limited training data. Therefore, overfitting occurs easily in the CE method owing to the limited training data. By contrast, our proposed method yields more accurate results than the CE method.

The contributions of our method are summarized as follows:

- We propose a new probability regularization method for limited training data using a self-knowledge distillation scheme;
- We propose a pixel-wise adaptive label smoothing (PALS) by fully utilizing the internal statistics of pixels within an input image;
- We demonstrate the effectiveness of our method by showing improved accuracy compared with previous methods for various ratios of training data, such as 10%, 30%, 50%, and 100% on the Cityscapes dataset [29] and the Pascal VOC2012 dataset [30].

## 2. Related Work

### 2.1. Semantic Segmentation

Semantic segmentation is a pixel-wise classification problem that aims to predict the categories of each pixel in a specified image. Various approaches have been proposed to improve the performance of semantic segmentation since the introduction of FCNs [6]. The encoder-decoder architecture [4,31,32] was proposed in early studies to recover spatial losses caused by pooling layers in the networks. Liu et al. [33] and Peng et al. [34] proposed enlarging the receptive field, which is crucial for obtaining context information. In addition to enlarging the receptive field and capturing multiscale context information, refs. [8,9,10,11] proposed pyramid feature pooling methods. To learn semantically richer and spatially more precise feature representations, [35,36,37,38,39,40] combined multiresolution feature maps. Based on the self-attention scheme [41,42], some researchers [43,44,45,46] proposed capturing relational context information by aggregating the relations between pixels.

However, because these studies did not consider situations involving limited training data, which are typically encountered in real-world applications, several researchers have proposed weakly/semi-supervised learning-based methods to address this issue. Refs. [47,48,49,50] used image-level labels, refs. [51,52,53] used bounding boxes, and [54,55,56,57,58,59,60] proposed utilizing unlabeled images. Whereas additional data or annotations are required in the above-mentioned methods, Zhao et al. [61] proposed a pretraining to address the problem of limited data. Specifically, they trained a network twice by pretraining a model based on label-based contrastive learning [62] first, and then fine-tuning the model with cross-entropy loss. Unlike the method described in [61], the proposed method does not require any pretraining.

### 2.2. Regularization

Regularization is a set of techniques that aims to avoid overfitting and improve the generalization of a model. Typical methods to avoid overfitting the training data include $L_{1}$/$L_{2}$-regularization [14], dropout [16], batch normalization [15] and data augmentation [63]. Additionally, some researchers have proposed regularizing the output of a model using target modification approaches. LS [20,21] uses soft targets, which are the weighted average of one-hot targets and uniform distribution over labels. CP [22,23] regularizes the output of a model by penalizing low-entropy output distributions. These methods prevent the model from becoming overconfident [20,22]. Recently, researchers have extended this idea to other tasks, such as domain adaptation [64,65], incremental learning [66], and self-knowledge distillation (self-KD) [24,67,68]. By contrast, this study focuses on training semantic segmentation models using a limited amount of labeled data. Additionally, the proposed method modifies the target distribution by aggregating the probabilities of pixels based on their similarities to the output of the model.

On the other hand, KD [25,69] exploits the predictions of the teacher model, which is relatively large, to transfer knowledge to the student model, which is relatively small. Recently, various approaches have been extended to semantic segmentation [70,71,72]. However, because the training process based on teacher-student knowledge distillation requires additional teacher networks, the computational costs are high. However, it has been demonstrated [24,67,68,73,74] that self-KD, which causes the model to learn knowledge from itself, is effective in exploiting a potential capacity of a single model. Although these works are simple and effective, they do not demonstrate the effectiveness of their works in the limited labeled data setting for semantic segmentation. Table 1 summarizes the strengths and weaknesses of the various regularization methods described above.

## 3. Revisit of CE, LS, CP, KD

In this section, we briefly describe previous distribution regularizing methods, including the CE loss function, LS [20,21], CP [22], and KD [25].

### 3.1. Cross Entropy

Since the introduction of the FCNs [6], most semantic segmentation networks have been designed using convolutional layers without fully connected layers. The features of the last convolutional layer in a model are known as logits $Z\in R^{C\times H^{\prime }\times W^{\prime }}$, where *C* is the number of classes, and $H^{\prime }$ and $W^{\prime }$ are the height and width of the logits, respectively. The predicted distribution map $\hat{P}\in R^{C\times H\times W}$ is then generated from *Z*, where *H* is the height, and *W* is the width of the original input image. It is noteworthy that when *Z* is different from $\hat{P}$ in terms of the spatial size, *Z* is typically resized to the same resolution as $\hat{P}$. In the typical setting, $\hat{P}$ is defined using the softmax operation, as follows:

(1) $${\hat{P}}_{c}\left(i\right)=\frac{\exp(Z_{c}\left(i\right))}{\sum_{c=1}^{C}\exp(Z_{c}\left(i\right))},$$

where ${\hat{P}}_{c}\left(i\right)$ denotes the probability of the *c*th channel of the *i*th pixel of $\hat{P}$. Subsequently, the CE loss $L_{CE}$ is defined as

(2) $$\begin{array}{c} L_{CE}\left(Y,\hat{P}\right)=\frac{1}{HW}\sum_{i=1}^{HW}H(Y\left(i\right),\hat{P}\left(i\right)),\;\;\\ H\left(Y\left(i\right),\hat{P}\left(i\right)\right)=-\frac{1}{C}\sum_{c=1}^{C}Y_{c}\left(i\right)\log\left({\hat{P}}_{c}\left(i\right)\right), \end{array}$$

where $Y\in R^{C\times H\times W}$ is a one-hot encoded distribution map using ground-truth labels, and $Y_{c}\left(i\right)$ is the value at the *c*th channel of the *i*th pixel of *Y*. $H(Y\left(i\right),\hat{P}\left(i\right))$ is the CE of the *i*th pixel. Typically, $L_{CE}$ is defined as the average CE value of for all pixels.

### 3.2. Label Smoothing

LS [20] adds a one-hot encoded ground truth and uniform distribution to generate a smooth probability distribution. Subsequently, the smoothed probability distribution map $Y_{s}\in R^{C\times H\times W}$ is defined as follows:

(3) $$Y_{s}\left(i\right)=\lambda U+\left(1-\lambda \right)Y\left(i\right),$$

where $Y_{s}\left(i\right)$ is the probability distribution vector of the *i*th pixel of $Y_{s}$, $U\in R^{C}$ is a uniform distribution vector, where each element is 1/C, and λ is the weighting factor for a uniform distribution vector. Subsequently, the label smoothing loss $L_{LS}$ is defined as

(4) $$L_{LS}\left(Y,\hat{P}\right)=\frac{1}{HW}\sum_{i=1}^{HW}H(Y_{s}\left(i\right),\hat{P}\left(i\right)).$$

### 3.3. Confidence Penalty

CP [22] induces an increase in the entropy of the predicted distribution $\hat{P}\left(i\right)$. The CP [22] loss $L_{CP}$ is defined as follows:

(5) $$L_{CP}\left(Y,\hat{P}\right)=\frac{1}{HW}\sum_{i=1}^{HW}H\left(Y\left(i\right),\hat{P}\left(i\right)\right)-\beta H\left(\hat{P}\left(i\right),\hat{P}\left(i\right)\right),\;\;$$

where β is a weighting factor, and $H(\hat{P}\left(i\right),\hat{P}\left(i\right))$ represents the entropy of $\hat{P}\left(i\right)$.

### 3.4. Knowledge Distillation

KD transfers the knowledge of a well-trained teacher network to the student network to improve its performance on the student network. Typically, the KD loss function $L_{KD}$ [25] is defined as

(6) $$\begin{array}{c} L_{KD}\left(Y,\hat{P}\right)=\frac{1}{HW}\sum_{i=1}^{HW}\gamma KL\left({\hat{P}}^{t}\left(i\right),\hat{P}\left(i\right)\right)+\left(1-\gamma \right)H\left(Y\left(i\right),\hat{P}\left(i\right)\right),\\ KL\left({\hat{P}}^{t}\left(i\right),\hat{P}\left(i\right)\right)=-\sum_{c=1}^{C}{\hat{P}}_{c}^{t}\left(i\right)\log\frac{{\hat{P}}_{c}\left(i\right)}{{\hat{P}}_{c}^{t}\left(i\right)}=H\left({\hat{P}}^{t}\left(i\right),\hat{P}\left(i\right)\right)-H\left({\hat{P}}^{t}\left(i\right),{\hat{P}}^{t}\left(i\right)\right), \end{array}$$

where ${\hat{P}}^{t}\left(i\right)$ denotes the predicted distribution of the teacher network at the *i*th pixel. KL(·,·) is the Kullback–Leibler (KL) divergence between the two distributions, and γ is a weighting value of the KL divergence term.

## 4. Proposed Method

In this section, we introduce our pixel-wise adaptive label smoothing (PALS) via self-knowledge distillation for semantic segmentation. We assume that only a small amount of training data are available to train the network. For each input image, various pixels share the same class, because one object comprises several pixels, and multiple objects may exist in an input image. Hence, our method generates a pixel-wise adaptive soft label for each pixel by aggregating the probability distributions of correctly estimated pixels of the same class. Soft labels function as a teacher in regularizing the distributions of each pixel.

**Figure 3:**
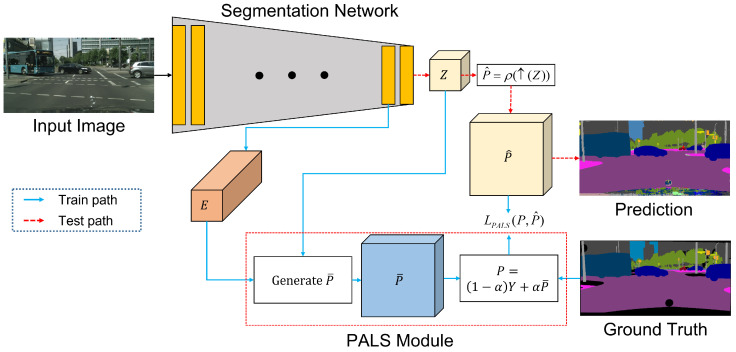
Overview of the proposed method, which is categorized into training and test paths. Blue and red arrows represent training and test paths, respectively.

Figure 3 shows an overview of our proposed method, which is categorized into training and test paths. Let an input image be $I\in R^{3\times H\times W}$, where *H* is the height, *W* is the width, and the number of color channels is three. In training path, to improve the network performance, we generate an adaptive soft label map $P\in R^{C\times H\times W}$ using the proposed PALS module, where *C* is the number of classes. The structure of the PALS module is explained in detail in the following subsection. By comparing *P* and the estimated probability distribution $\hat{P}\in R^{C\times H\times W}$, we compute a loss for training the network. In the test path, we predict our result using only the probability distribution $\hat{P}$.

### 4.1. PALS Module

**Figure 4:**
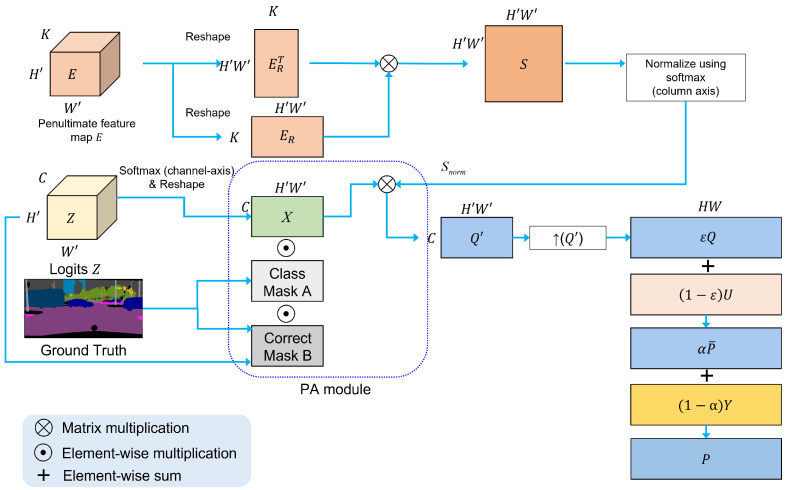
Process of our PALS module.

Figure 4 illustrates our PALS module. The input features of the module are logits $Z\in R^{C\times H^{\prime }\times W^{\prime }}$ and penultimate feature map $E\in R^{K\times H^{\prime }\times W^{\prime }}$, where *K* is the number of channels of the penultimate feature map, and $H^{\prime }$ and $W^{\prime }$ are the spatial sizes. To compute a similarity matrix $S\in R^{H^{\prime }W^{\prime }\times H^{\prime }W^{\prime }}$ that contains similarities or correlations between all pairs of features in *E*, we perform matrix multiplication using reshaped matrices $E_{R}\in R^{K\times H^{\prime }W^{\prime }}$ and $E_{R}^{T}\in R^{H^{\prime }W^{\prime }\times K}$ from *E*. Therefore, *S* is defined as

(7) $$S=E_{R}^{T}\cdot E_{R}=\left[s_{1},s_{2},\cdots ,s_{H^{\prime }W^{\prime }}\right],$$

where $s_{i}\in R^{H^{\prime }W^{\prime }\times 1}$ is a column vector that includes all correlations between a feature of the *i*th spatial position and all features in *E*. To perform normalization for each column vector, we performed a softmax operation along each column axis. Subsequently, $S_{norm}$ is defined as

(8) $$S_{norm}=\left[\rho \left(s_{1}\right),\rho \left(s_{2}\right),\cdots ,\rho \left(s_{H^{\prime }W^{\prime }}\right)\right],$$

where ρ(·) represents the softmax operation.

**Figure 5:**
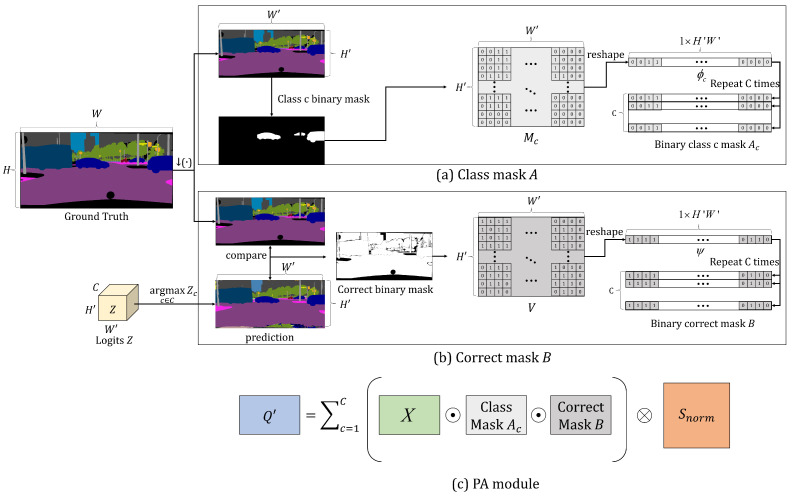
Process of PA module, where ↓(·) denotes the downsampling operation.

To compensate for insufficient training data, we fully utilize the internal statistics of the pixels in the input image. In this regard, we compute a pixel-wise ensemble of distributions by adaptively aggregating the distributions of other pixels based on the pixel affinity. Thus, we have generated an aggregated distribution map $Q^{\prime }\in R^{C\times H^{\prime }W^{\prime }}$ from a proposed probability aggregation (PA) module, which exploits the information of correctly estimated pixels of the same class in an input image. Figure 5 shows the process of the PA module in detail. To compute $Q^{\prime }$, we generate a set of class masks $A={\left\{A_{c}\in R^{C\times H^{\prime }W^{\prime }}\right\}}_{c=1,2,\ldots C}$ and a correct mask *B*. To generate a class mask $A_{c}$ that corresponds to the *c*th class, we create a binary mask $M_{c}\in R^{H^{\prime }\times W^{\prime }}$ for the *c*th class using a downsampled ground-truth image. An element of $M_{c}$ in each spatial position has a value of 1 when the ground-truth label corresponds to class *c*, and 0 otherwise. Furthermore, we reshape $M_{c}$ to generate a one-dimensional vector $\phi_{c}\in R^{1\times H^{\prime }W^{\prime }}$. Subsequently, we concatenate the $\phi_{c}$ vector *C* times along the column axis to generate $A_{c}$. However, to create a correct mask $B\in R^{C\times H^{\prime }W^{\prime }}$, we generate a binary map $V\in R^{H^{\prime }\times W^{\prime }}$, where each element of *V* is 1 when the predicted label using *Z* is correct, and 0 otherwise. We reshape *V* to generate $\psi \in R^{1\times H^{\prime }W^{\prime }}$. Subsequently, the correct mask *B* is obtained by concatenating the ψ vector *C* times along column axis. Subsequently, $Q^{\prime }$ and $Q\in R^{C\times H\times W}$ are defined as

(9) $$\begin{array}{cc} \; & Q^{\prime }=\sum_{c=1}^{C}\left(X⊙A_{c}⊙B\right)⊗S_{norm},\\ \; & Q=↑(Q^{\prime }), \end{array}$$

where ⊙ is an element-wise multiplication operation, and ⊗ is a matrix multiplication operation. ↑(·) is an upsampling operation that uses a bilinear interpolation. $X\in R^{C\times H^{\prime }W^{\prime }}$ is a probability distribution map obtained by performing the softmax operation along the channel axis for each pixel from *Z* and then reshaping it. *Q* is the upsampled result of $Q^{\prime }$.

It is noteworthy that the aggregated distribution *Q* at the early iteration is not sufficiently accurate, because only a few pixels are correct in the early iteration. Hence, we adaptively combined *Q* and the uniform distribution *U* as a function of the current iteration number τ. Subsequently, the fused probability distribution map ${\bar{P}}_{\tau }\in R^{C\times H\times W}$ at iteration τ is defined as

(10) $${\bar{P}}_{\tau }\left(i\right)=\varepsilon Q\left(i\right)+\left(1-\varepsilon \right)U,\;\;\left(\varepsilon =\frac{\tau }{T}\right),$$

where ${\bar{P}}_{\tau }\left(i\right)$ is the distribution vector at the *i*th pixel in ${\bar{P}}_{\tau }$. *U* is a uniform distribution vector where each element is 1/C. T is the total iteration number, and τ is the current iteration number. Here, ε represents the ratio of the current iteration τ to the total iterations T, similar to [75].

Generally, aggregated distribution and one-hot encoded distributions exhibit different properties. The former reduces the variance error, whereas the latter reduces the bias error [28]. Therefore, we combined ${\bar{P}}_{\tau }$ and a one-hot encoded distribution map $Y\in R^{C\times H\times W}$ to reap the advantages of both and then generated the final soft label $P_{\tau }\in R^{C\times H\times W}$, called a pixel-wise adaptive label smoothing (PALS). Here, $P_{\tau }\left(i\right)$, the probability distribution vector of the *i*th pixel in $P_{\tau }$ at iteration τ is defined as

(11) $$P_{\tau }\left(i\right)=\alpha {\bar{P}}_{\tau }\left(i\right)+\left(1-\alpha \right)Y\left(i\right),$$

where Y(i) is a one-hot vector at the *i*th pixel in *Y*, and α is the weighting factor between two vectors ${\bar{P}}_{\tau }\left(i\right)$ and Y(i). It is noteworthy that, at the initial iteration, where ε is 0, $P_{\tau }\left(i\right)$ is the same as $Y_{s}\left(i\right)$ in Equation (3). As iteration progresses, ε increases up to 1, and the uniform distribution *U* in Equation (3) is replaced with the pixel-wise aggregated probability *Q* in Equation (9).

### 4.2. Loss Function

The loss function $L_{PALS}$ for training the network is defined as

(12) $$L_{PALS}\left(P_{\tau },{\hat{P}}_{\tau }\right)=\frac{1}{HW}\sum_{i=1}^{HW}KL(P_{\tau }\left(i\right),{\hat{P}}_{\tau }\left(i\right)),$$

where $P_{\tau }\left(i\right)$ and ${\hat{P}}_{\tau }\left(i\right)$ are the proposed soft target defined in Equation (11) and the predicted distribution of the *i*th pixel at iteration τ, respectively. We computed our loss function using the KL divergence between the two distributions.

## 5. Experiments

In this section, we compare our proposed method with previous methods and analyze the effectiveness of our proposed method based on various experimental settings. Further details are provided in the following subsections.

### 5.1. Dataset

To perform evaluations, we used the Cityscapes [29] dataset and the Pascal VOC2012 [30] dataset for semantic segmentation. The Cityscapes dataset includes urban scenes for semantic segmentation, and it contains 30 classes; however, we used only 19 classes for training and testing, similar to previous studies [9,10,11]. Each image exhibited a high resolution of 2048×1024. The dataset contains 5000 pixel-level finely annotated images and 20,000 coarsely annotated images. In the finely annotated images, 2975/500/1525 images are allocated for training, validation, and testing, respectively. We used only finely annotated images for training. The Pascal VOC2012 dataset [30] is one of the most competitive semantic segmentation datasets. It contains 21 classes, including 20 foreground classes and 1 background class. This dataset consists of 10,582 training, 1449 validation, and 1456 test images.

### 5.2. Implementation Details

Our method was applied to the DeepLab-V3+ [10] model, with Xception65 [76] and ResNet18 [77] as backbone networks. The former is a deeper and heavier network than the latter. We initialized the backbone networks using weights pretrained on the ImageNet [78] dataset, whereas the weights of other modules, such as the ASPP module [10], were randomly initialized. To train the networks, we set the initial learning rate to 0.02, and we used the polynomial learning rate scheduler with factor $\left(1-{\left(\frac{\tau }{T}\right)}^{0.9}\right)$ using SGD optimization. For unbiased comparisons, we used the same hyperparameters, including a batch size of 8, and 200 epochs for all the experiments. For the Cityscapes dataset, to evaluate the accuracy of the networks for a limited amount of training data, we randomly selected 10%, 30%, 50%, and 100% of the images from the original training dataset, where each proportion comprises 297, 894, 1487, and 2975 training images, respectively. For data augmentation, we performed random horizontal flipping and random-scale cropping. The random scale range was (0.5, 2.0), and the cropping size was 384 × 384. During training, half-size images were used to reduce memory consumption, and full-size images were used on the validation and test data after the results were upsampled. To identify a suitable weighting factor α in Equation (11), we performed several experiments by changing α to {0.05,0.1,0.15,0.2,0.25,0.3,0.4,0.5}, and empirically discovered that α=0.2 yielded the best results; hence, we used it for all the experiments.

For the Pascal VOC2012 dataset [30], we set mostly the same parameters as those of the Cityscapes dataset except the cropping size and the number of training epochs. Our method was applied to the DeepLab-V3+ [10] with Xception65 [76] as the backbone network for the Pascal VOC2012 dataset. The cropping size was set at 480 × 480, and the number of training epochs was 100. We randomly selected 10%, 30%, 50%, and 100% of the images from the original training set, where each proportion comprises 1059, 3175, 5291, and 10,582 training images, respectively.

### 5.3. Comparison with Previous Methods

We compared our proposed method with previous methods, including CE [10], LS [20], and CP [22]. For LS [20] and CP [22], we empirically determined the values of λ in Equation (3) and β in Equation (5) that the best performance was achieved using λ=0.2 and β=0.1. For unbiased comparisons, we used the same limited training data for all the comparative methods.

#### 5.3.1. The Cityscapes Dataset

Table 2 lists the mIoU results of the Cityscapes training, validation, and test data for DeepLab-V3+ with the Xception65 network.

Each column represents the data ratio used in the training data, and each row represents a different method. Each method was trained three times, and the average values of the mIoU and corresponding variances are listed in Table 2. It is noteworthy that all methods suffer from overfitting when the amount of training data were sufficiently small. The accuracy for the training data was favorable, whereas that of the validation and test data decreased significantly. The results for the validation data show that our method yielded the best accuracy, except for the results based on only 10% of the data. Meanwhile, based on the results of the test data, our method show the best accuracy for all data ratios as compared with the other methods. LS generates soft labels by adding a uniform distribution to a one-hot vector, which results in a better accuracy than the baseline CE method. However, LS is suboptimal for the regularization function because it does not consider the correlation between classes. CP regularizes the distribution by subtracting its entropy. CP performs worse as the amount of training data decreases because the entropy of the distribution is already large, particularly when the training data are limited.

**Figure 6:**
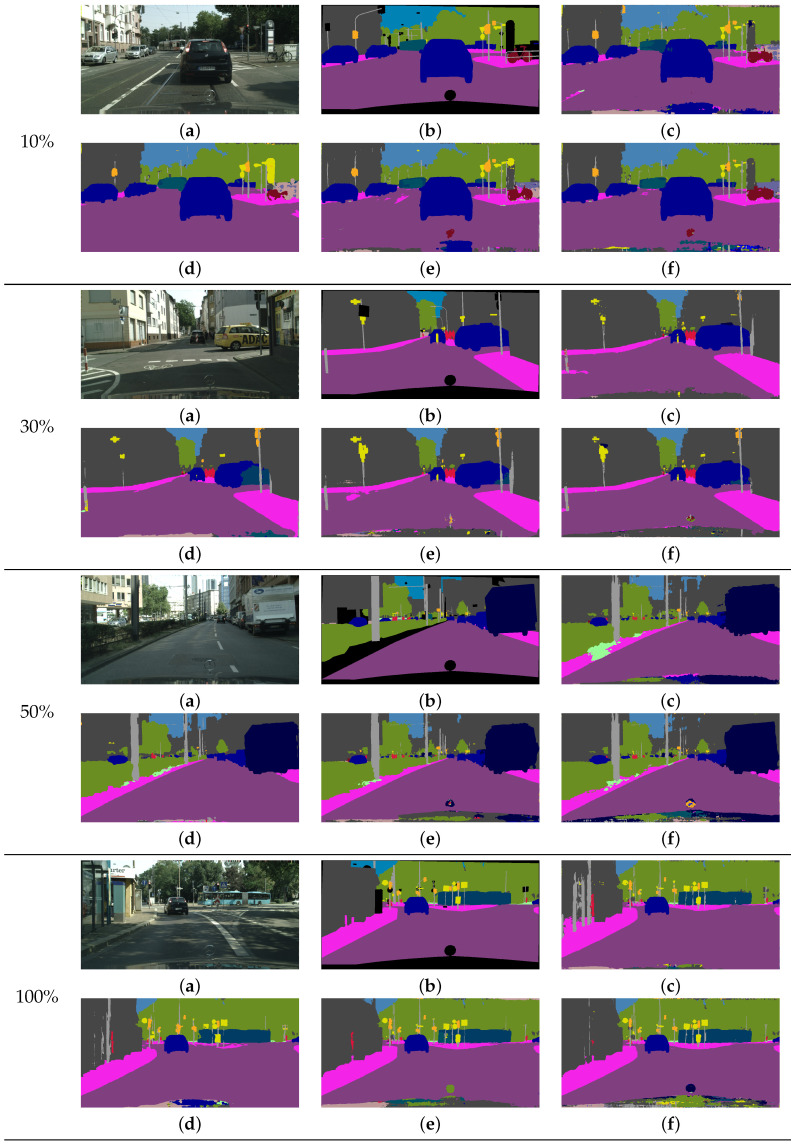
Results of the comparison of various methods using limited training data for DeepLab-V3+ [10] with the Xception65 [76] network on the Cityscapes dataset. (**a**) Input image. (**b**) Ground-truth image. (**c**) CE [10] result. (**d**) CP [22] result. (**e**) LS [20] result. (**f**) Our result.

Figure 6 shows the qualitative comparison results of different methods for DeepLab-V3+ [10] with the Xception65 [76] network on the validation data. The ratio numbers in the first column in Figure 6 denote the data ratios used for training from the original training set. It is observed that our method generates results with more accurate boundary regions and less noise for homogeneous regions in the train, truck, and bus objects compared with other methods. For the 10% training data, the results of most methods include severe errors and ambiguous boundaries for the pole and bus classes, which contain fewer labeled pixels than the other classes. By contrast, our method yields more accurate and clearer boundaries for those classes. Similarly, for the 30% training data, our method yields less noise for the object boundaries of cars and buildings as compared with the other methods. For the 50% training data, our method predicts the boundaries of trucks more clearly as compared with other methods. For the 100% training data, our method yields predictions that are better than those of LS [20] and CP [22] for the bus objects, and better than that of CE [10] for the buildings.

Table 3 shows the mIoU results of various methods for DeepLab-V3+ [10] with the ResNet18 backbone [77], which is a lighter networks than DeepLab-V3+ [10] with the Xception65 backbone [76]. Table 3 shows that our method achieves the best accuracy, except for the results based on only 100% validation data and 50% test data. As shown in Table 3, LS performs better than CE for most cases, whereas CP [22] is less accurate than CE [10] for the 10% and 50% validation and test data, respectively. This is because a light network typically exhibits lower confidence in term of probability distribution compared with a heavy network [79]. Therefore, CP [22] resulted in reduced accuracy because it enlarged the entropy of the probability distributions.

**Figure 7:**
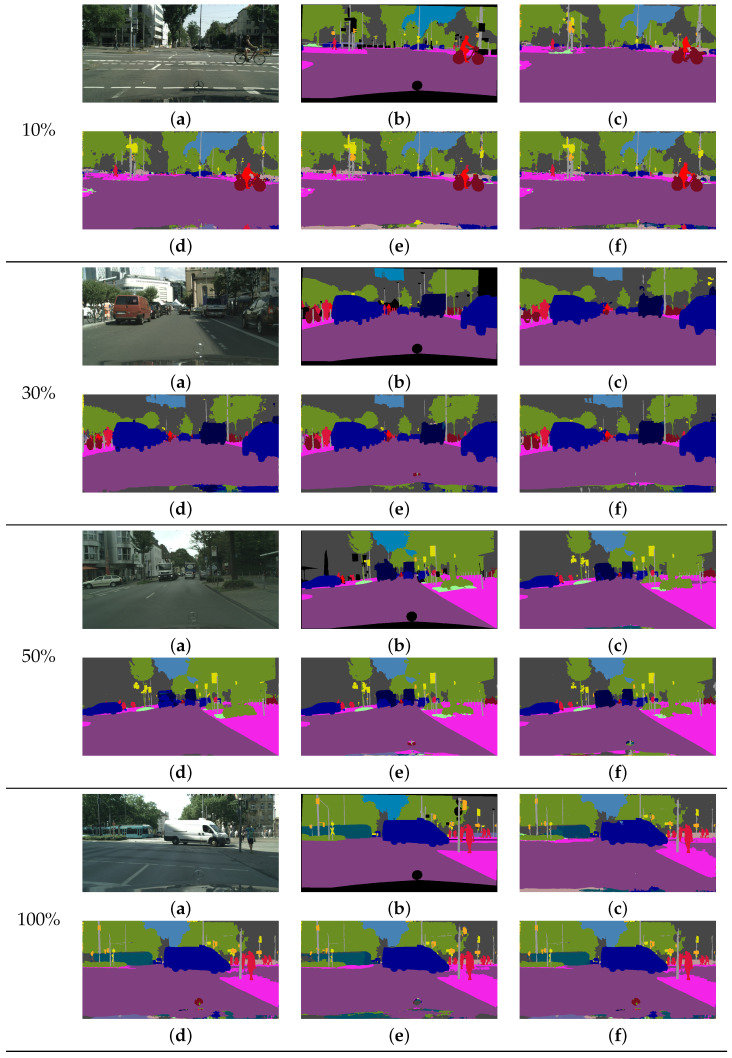
Results of the comparison of various methods using limited training data for DeepLab-V3+ [10] with the ResNet18 [77] network on the Cityscapes dataset. (**a**) Input image. (**b**) Ground-truth image. (**c**) CE [10] result. (**d**) CP [22] result. (**e**) LS [20] result. (**f**) Our result.

Figure 7 shows the qualitative comparison results of different methods using DeepLab-V3+ [10] with the ResNet18 [77] network on the validation data. Because a light network was used, these results indicate less accurate performance than the heavy network. However, our results show clearer boundaries and less noise compared with the other methods. For the 10% training data, our method yielded better predictions than the other methods for rider objects. For the 30% and 50 % training data, our method yielded more accurate results, particularly for truck objects, compared with the other methods. For the 100% training data, our method yielded better predictions for train objects compared with the other methods.

#### 5.3.2. Pascal VOC2012 Dataset

Table 4 shows the mIoU results of various methods for DeepLab-V3+ [10] with the Xception65 network [76] on the Pascal VOC2012 dataset. It is observed that our method achieves the best accuracy for all the cases in validation and test data. Specifically, our method achieved mIoU improvements of 1.447%, 0.713%, 3.185%, and 1.153% for 10%, 30%, 50%, and 100% training data compared with the baseline method, respectively. LS performs better than CE for all cases, whereas CP [22] is less accurate than CE [10], except when using 100% training data.

**Figure 8:**
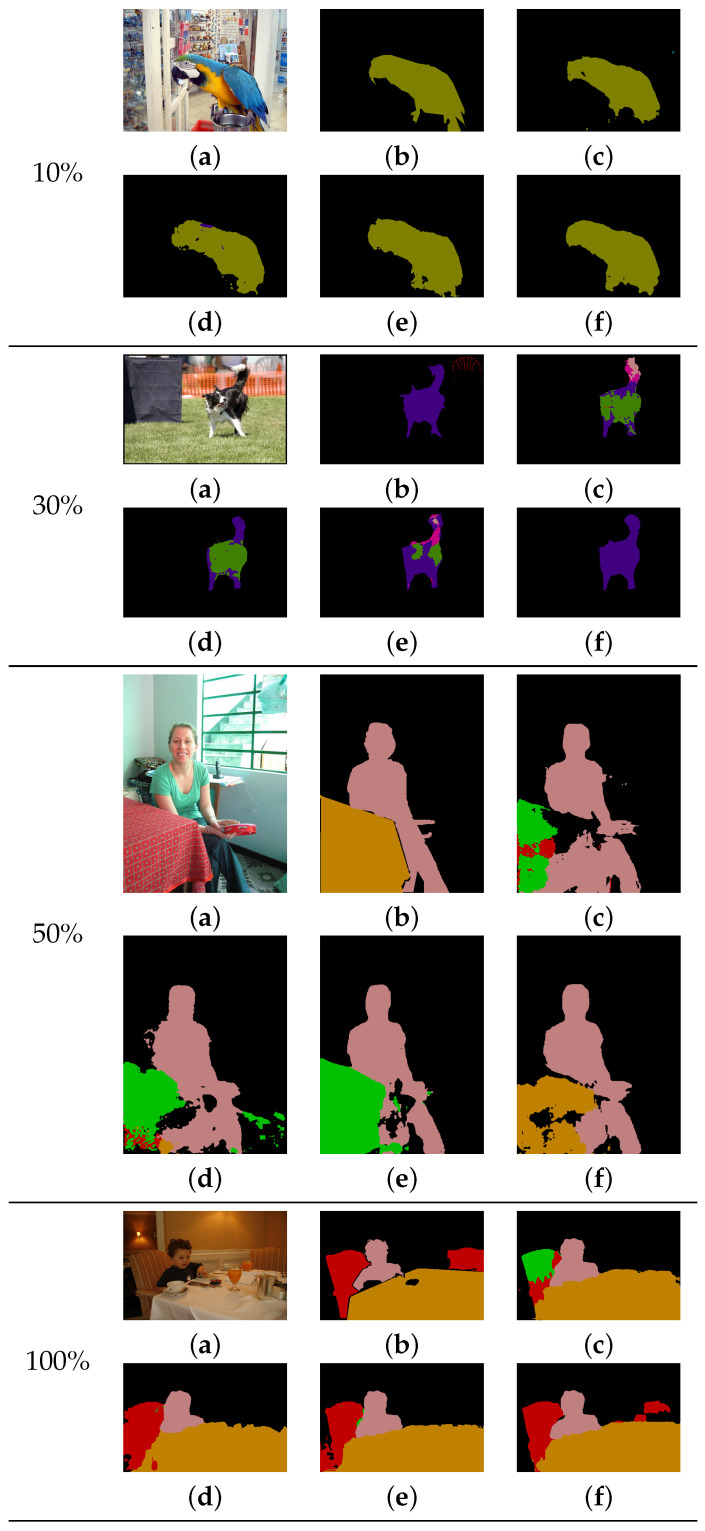
Results of the comparison of various methods using limited training data for DeepLab-V3+ [10] with the Xception65 [76] network on the Pascal VOC2012 dataset. (**a**) Input image. (**b**) Ground-truth image. (**c**) CE [10] result. (**d**) CP [22] result. (**e**) LS [20] result. (**f**) Our result.

Figure 8 shows the qualitative comparison results of different methods using DeepLab-V3+ [10] with the Xception65 [76] network on the validation data. Since our method aggregates distributions using correctly estimated pixels based on the pair-wise feature similarity, the objects in our results have more accurate boundaries and less noise compared with other methods that independently estimate each pixel. For the 10% training data, for example, the predicted result for the bird class of our method is more accurate than others. For the 30% and 50% training data, most other methods incorrectly predict the dog class and the table class, respectively. In contrast, our method correctly estimates them. For the 100% training data, our method yields better predictions for a complex table class consisting of a multitude of small objects compared with other methods.

## 6. Ablation Study

As introduced in Section 4, our proposed method generates a pixel-wise adaptive soft label *P* in Equation (11) and uses it to define a loss function. When generating *P*, we used multiple components, including the same-class masks *A*, the correct mask *B*, a uniform distribution *U*, and an adaptive weight ε. To investigate the effectiveness of our proposed method, we conducted experiments where each component was removed from our original method. Table 5 shows the mIoU values obtained when each component was removed from our original method. The first row shows the mIoU results of our original method, defined by Equation (11). Here, “without class mask *A*” represents a method where the mask *A* is removed in Equation (9) by setting all elements in *A* to 1. Furthermore, “without correct mask *B*” represents a method where the mask *B* is removed in Equation (9) by setting all the elements in *B* to 1, and “without uniform distribution *U*” represents a method where the uniform distribution *U* is removed from Equation (10) by setting the value of ε to 1 for all the iterations. Lastly, “without adaptive weight ε” represents a method that fixes the weight ε to 0.5 in Equation (10) for all the iterations instead of using it in an adaptive manner.

By comparing the results presented in the first and second rows in Table 5, the effectiveness of using *A* was observed. When pixels of different classes participated in the computation of *Q*, the probability distributions of the pixels were mixed with those of the other classes, which resulted in less accurate results. By comparing the results of the first and third rows in Table 5, the effectiveness of using *B* was observed. When the incorrectly estimated pixels participated in the computation of *Q*, the erroneous probability distributions of the pixels contaminated the final soft targets, which resulted in less accurate results. By comparing the first and fourth row results in Table 5, the effectiveness of using the uniform distribution *U* was observed. In the early iteration of the training step, the aggregated distribution *Q* was inaccurate because many pixels were incorrect pixels. Hence, a uniform distribution *U* is more beneficial than *Q* in the early iterations. The effectiveness of the adaptive weight ε was investigated by comparing the first and last rows in Table 5. If we fix the value of ε to 0.5, then the weights of *Q* and *U* will be the same for all iterations. Because *Q* contains reliable information in the later iterations, it cannot fully function adaptively when ε is fixed.

On the other hand, to investigate the effect of varying α in Equation (11), we performed several experiments by changing α values to {0.05, 0.1, 0.15, 0.2, 0.25, 0.3, 0.4, 0.5}. Table 6 shows the mIoU values of the proposed method on the Cityscapes validation data using DeepLab-V3+ [10] with the Xception65 network [76] as a function of the α values. It is observed that our method generates similar performances for various α values for most cases. When α is 0.2, our method generates the best performance, except for the 50% dataset case. Based on these results, we fixed α at 0.2 for all the comparative evaluations.

## 7. Conclusions

We have proposed a pixel-wise adaptive label smoothing (PALS) method via self-knowledge distillation to train semantic segmentation networks for limited training data. In this regard, we aggregated the distribution of each pixel to fully utilize redundant information in an image by computing a similarity matrix that encodes the correlations between pairs of pixels. Based on the similarity matrix, we proposed a soft label by progressively adding a one-hot encoded label and the aggregated distribution for each pixel as a function of iteration. Our method yielded the most accurate results for various ratios of limited training data on the Cityscapes dataset and the Pascal VOC2012 dataset compared with previous regularization methods using DeepLab-V3+ with the Xception65 and ResNet18 networks.

## Figures and Tables

**Table 1:** Strengths and weaknesses of various regularization methods.

Method	Strength	Weakness
LS [20]	It has a positive effect on generalization using the weighted sum of one-hot encoding and the uniform distribution; It can be applied when a teacher model is not available.	The weighting factor for the uniform distribution is fixed and not learnable; The uniform distribution is not learnable and is not optimal for each pixel.
CP [22]	It has a positive effect on generalization using the entropy term; It can be applied when a teacher model is not available.	The weighting factor for the entropy term is fixed and not learnable; It may increase the ambiguity of the estimated distribution when the entropy of the distribution is already large.
KD [25]	It has a positive effect on generalization by use of the prediction of the teacher network.	It cannot be applied when a teacher model is not available.
Ours	It has a positive effect on generalization using the weighted sum of one-hot encoding and the pixel-wise aggregated distribution; It can be applied when a teacher model is not available.	The weighting factor for the pixel-wise aggregated distribution is fixed and not learnable.

**Table 2:** mIoU values of different methods for DeepLab-V3+ with the Xception65 network. Bold expressions indicate the best accuracy.

Method	Data	10%	30%	50%	100%
CE (baseline) [10]	train	79.054 ± 0.307	81.650 ± 0.511	82.291 ± 0.024	82.586 ± 0.157
	val	**59.886 ± 0.430**	67.756 ± 0.312	69.895 ± 0.212	73.167 ± 0.155
	test	59.348 ± 0.046	66.224 ± 1.270	69.522 ± 0.003	72.272 ± 0.237
LS [20]	train	78.117 ± 0.040	82.032 ± 0.001	83.505 ± 0.008	83.219 ± 0.117
	val	59.459 ± 0.051	68.822 ± 0.141	70.190 ± 0.499	73.748 ± 0.137
	test	59.331 ± 0.015	67.717 ± 0.111	69.606 ± 0.081	72.542 ± 0.082
CP [22]	train	76.269 ± 13.697	79.411 ± 6.892	80.755 ± 4.305	82.303 ± 2.204
	val	58.137 ± 3.820	67.715 ± 4.373	70.517 ± 0.393	73.830 ± 0.151
	test	57.339 ± 3.997	65.397 ± 1.011	68.650 ± 0.483	72.814 ± 0.643
Ours	train	78.641 ± 0.187	81.784 ± 0.799	82.711 ± 0.002	83.342 ± 0.023
	val	59.767 ± 0.209	**69.285 ± 0.618**	**70.974 ± 0.240**	**73.889 ± 0.288**
	test	**59.424 ± 0.122**	**68.072 ± 0.077**	**70.659 ± 0.467**	**73.335 ± 0.102**

**Table 3:** mIoU values of different methods for DeepLab-V3+ with the ResNet18 network. Bold expressions indicate the best accuracy.

Method	Data	10%	30%	50%	100%
CE (baseline) [10]	train	68.878 ± 1.464	74.616 ± 2.380	76.337 ± 5.025	78.619 ± 0.061
	val	51.215 ± 2.327	61.104 ± 2.133	63.656 ± 2.274	67.754 ± 3.187
	test	51.091 ± 0.873	59.862 ± 1.684	63.506 ± 3.966	68.795 ± 0.213
LS [20]	train	70.774 ± 1.395	78.384 ± 0.195	78.766 ± 0.020	79.837 ± 0.025
	val	53.650 ± 0.736	64.088 ± 0.038	65.463 ± 0.050	**70.424 ± 0.087**
	test	54.182 ± 0.508	62.089 ± 0.048	**65.507 ± 0.059**	69.752 ± 0.139
CP [22]	train	66.839 ± 34.734	74.860 ± 1.199	75.303 ± 1.723	78.585 ± 0.263
	val	49.267 ± 12.073	61.485 ± 1.302	63.292 ± 1.325	69.134 ± 0.120
	test	49.943 ± 10.104	60.262 ± 1.324	62.889 ± 2.235	69.752 ± 0.139
Ours	train	71.452 ± 1.704	78.227 ± 0.113	78.838 ± 0.131	79.849 ± 0.003
	val	**54.219 ± 0.065**	**64.172 ± 0.157**	**65.672 ± 0.005**	70.374 ± 0.008
	test	**54.683 ± 0.048**	**62.649 ± 0.022**	65.441 ± 0.175	**69.837 ± 0.118**

**Table 4:** mIoU values of different methods for DeepLab-V3+ with Xception65 network on the Pascal VOC2012 dataset [30]. Bold expressions indicate the best accuracy.

Method	Data	10%	30%	50%	100%
CE (baseline) [10]	val	57.338	67.049	70.079	74.708
	test	56.538	68.240	69.745	73.615
LS [20]	val	56.981	69.772	73.733	76.320
	test	57.111	68.666	72.535	74.650
CP [22]	val	52.951	68.424	69.603	74.373
	test	53.982	67.368	69.452	73.817
Ours	val	**58.989**	**70.939**	**73.814**	**76.407**
	test	**57.985**	**68.953**	**72.930**	**74.768**

**Table 5:** Results of DeepLab-V3+ [10] with the Xception65 [76] on the Cityscapes validation data. “w/o X” represents a method where component of “X” was removed from our original method. Bold expressions indicate the best accuracy.

Method	10%	30%	50%	100%
Our original method	**60.279**	**69.912**	**71.535**	**73.849**
w/o class mask *A*	59.181	68.756	71.317	73.490
w/o correct mask *B*	59.211	69.620	70.706	73.369
w/o uniform distribution *U*	59.785	69.107	71.279	72.563
w/o adaptive weight ε	59.902	68.681	71.481	72.996

**Table 6:** mIoU values of our method by varying α values on the Cityscapes validation data. The value of α represents the weighting factor in Equation (11). Bold expressions indicate the best accuracy.

α	10%	30%	50%	100%
0.05	59.552	68.080	69.352	73.153
0.10	60.074	68.399	70.269	72.686
0.15	60.201	66.600	68.719	73.093
0.20	**60.279**	**69.912**	71.535	**73.849**
0.25	59.758	69.683	**71.830**	73.540
0.30	59.807	69.481	69.780	73.450
0.40	58.150	68.095	71.040	72.629
0.50	58.062	69.460	70.531	73.746

## Data Availability

Data sharing not applicable.

## References

[1] Zeng W., Luo W., Suo S., Sadat A., Yang B., Casas S., Urtasun R. End-To-End Interpretable Neural Motion Planner. Proceedings of the IEEE Conference on Computer Vision and Pattern Recognition (CVPR).

[2] Philion J., Fidler S. Lift, Splat, Shoot: Encoding Images From Arbitrary Camera Rigs by Implicitly Unprojecting to 3D. Proceedings of the European Conference on Computer Vision (ECCV).

[3] Cherabier I.F., Schönberger J.L., Oswald M.R., Pollefeys M., Geiger A. Learning Priors for Semantic 3D Reconstruction. Proceedings of the European Conference on Computer Vision (ECCV).

[4] Ronneberger O., Fischer P., Brox T. U-Net: Convolutional Networks for Biomedical Image Segmentation. Proceedings of the Medical Image Computing and Computer-Assisted Intervention (MICCAI).

[5] Srivastava A., Jha D., Chanda S., Pal U., Johansen H.D., Johansen D., Riegler M.A., Ali S., Halvorsen P. (2021). MSRF-Net: A Multi-Scale Residual Fusion Network for Biomedical Image Segmentation. arXiv.

[6] Long J., Shelhamer E., Darrell T. Fully convolutional networks for semantic segmentation. Proceedings of the IEEE Conference on Computer Vision and Pattern Recognition (CVPR).

[7] Chen L.C., Papandreou G., Kokkinos I., Murphy K., Yuille A.L. Semantic Image Segmentation with Deep Convolutional Nets and Fully Connected CRFs. Proceedings of the International Conference on Learning Representations (ICLR).

[8] Chen L., Papandreou G., Kokkinos I., Murphy K., Yuille A.L. (2018). DeepLab: Semantic Image Segmentation with Deep Convolutional Nets, Atrous Convolution, and Fully Connected CRFs. IEEE Trans. Pattern Anal. Mach. Intell..

[9] Chen L., Papandreou G., Schroff F., Adam H. (2017). Rethinking Atrous Convolution for Semantic Image Segmentation. arXiv.

[10] Chen L.C., Zhu Y., Papandreou G., Schroff F., Adam H. Encoder-Decoder with Atrous Separable Convolution for Semantic Image Segmentation. Proceedings of the European Conference on Computer Vision.

[11] Zhao H., Shi J., Qi X., Wang X., Jia J. Pyramid Scene Parsing Network. Proceedings of the IEEE Conference on Computer Vision and Pattern Recognition (CVPR).

[12] Dubey A., Gupta O., Raskar R. Regularizing Prediction Entropy Enhances Deep Learning with Limited Data. Proceedings of the Neural Information Processing Systems (NIPS).

[13] Bishop C. Regularization and complexity control in feed-forward networks. Proceedings of the International Conference on Artificial Neural Networks ICANN’95.

[14] Nowlan S.J., Hinton G.E. (1992). Simplifying Neural Networks by Soft Weight-Sharing. Neural Comput..

[15] Ioffe S., Szegedy C. Batch Normalization: Accelerating Deep Network Training by Reducing Internal Covariate Shift. Proceedings of the 32nd International Conference on Machine Learning.

[16] Srivastava N., Hinton G., Krizhevsky A., Sutskever I., Salakhutdinov R. (2014). Dropout: A Simple Way to Prevent Neural Networks from Overfitting. J. Mach. Learn. Res..

[17] Zhang H., Cisse M., Dauphin Y.N., Lopez-Paz D. mixup: Beyond Empirical Risk Minimization. Proceedings of the International Conference on Learning Representations (ICLR).

[18] Yun S., Han D., Oh S.J., Chun S., Choe J., Yoo Y. CutMix: Regularization Strategy to Train Strong Classifiers with Localizable Features. Proceedings of the IEEE International Conference on Computer Vision (ICCV).

[19] DeVries T., Taylor G.W. (2017). Improved Regularization of Convolutional Neural Networks with Cutout. arXiv.

[20] Müller R., Kornblith S., Hinton G.E. When does label smoothing help? In Proceedings of the Advances in Neural Information Processing Systems, Vancouver, BC, Canada, 8–14 December 2019; Volume 32.

[21] Szegedy C., Vanhoucke V., Ioffe S., Shlens J., Wojna Z. Rethinking the Inception Architecture for Computer Vision. Proceedings of the IEEE Conference on Computer Vision and Pattern Recognition (CVPR).

[22] Pereyra G., Tucker G., Chorowski J., Kaiser L., Hinton G.E. Regularizing Neural Networks by Penalizing Confident Output Distributions. Proceedings of the International Conference on Learning Representations (ICLR), OpenReview.net.

[23] Dubey A., Gupta O., Raskar R., Naik N. Maximum-Entropy Fine Grained Classification. Proceedings of the Advances in Neural Information Processing Systems.

[24] Yun S., Park J., Lee K., Shin J. Regularizing Class-Wise Predictions via Self-Knowledge Distillation. Proceedings of the IEEE Conference on Computer Vision and Pattern Recognition (CVPR).

[25] Hinton G., Vinyals O., Dean J. (2015). Distilling the Knowledge in a Neural Network. arXiv.

[26] Gou J., Yu B., Maybank S.J., Tao D. (2021). Knowledge Distillation: A Survey. Int. J. Comput. Vis..

[27] Hoffman J., Tzeng E., Darrell T., Saenko K. Simultaneous Deep Transfer Across Domains and Tasks. Proceedings of the IEEE International Conference on Computer Vision (ICCV).

[28] Zhou H., Song L., Chen J., Zhou Y., Wang G., Yuan J., Zhang Q. Rethinking Soft Labels for Knowledge Distillation: A Bias–Variance Tradeoff Perspective. Proceedings of the International Conference on Learning Representations (ICLR).

[29] Cordts M., Omran M., Ramos S., Rehfeld T., Enzweiler M., Benenson R., Franke U., Roth S., Schiele B. The Cityscapes Dataset for Semantic Urban Scene Understanding. Proceedings of the IEEE Conference on Computer Vision and Pattern Recognition (CVPR).

[30] Everingham M., Van Gool L., Williams C.K.I., Winn J., Zisserman A. The PASCAL Visual Object Classes Challenge 2012 (VOC2012) Results. http://www.pascal-network.org/challenges/VOC/voc2012/workshop/index.html.

[31] Noh H., Hong S., Han B. Learning Deconvolution Network for Semantic Segmentation. Proceedings of the IEEE International Conference on Computer Vision (ICCV).

[32] Badrinarayanan V., Kendall A., Cipolla R. (2017). SegNet: A Deep Convolutional Encoder-Decoder Architecture for Image Segmentation. IEEE Trans. Pattern Anal. Mach. Intell..

[33] Liu W., Rabinovich A., Berg A.C. (2015). Parsenet: Looking wider to see better. arXiv.

[34] Peng C., Zhang X., Yu G., Luo G., Sun J. Large Kernel Matters—Improve Semantic Segmentation by Global Convolutional Network. Proceedings of the IEEE Conference on Computer Vision and Pattern Recognition (CVPR).

[35] Zhao H., Qi X., Shen X., Shi J., Jia J. ICNet for Real-Time Semantic Segmentation on High-Resolution Images. Proceedings of the European Conference on Computer Vision (ECCV).

[36] Lin G., Milan A., Shen C., Reid I. RefineNet: Multi-Path Refinement Networks for High-Resolution Semantic Segmentation. Proceedings of the IEEE Conference on Computer Vision and Pattern Recognition (CVPR).

[37] Wang J., Sun K., Cheng T., Jiang B., Deng C., Zhao Y., Liu D., Mu Y., Tan M., Wang X. (2021). Deep High-Resolution Representation Learning for Visual Recognition. IEEE Trans. Pattern Anal. Mach. Intell..

[38] Xiao T., Liu Y., Zhou B., Jiang Y., Sun J. Unified Perceptual Parsing for Scene Understanding. Proceedings of the European Conference on Computer Vision (ECCV).

[39] Li X., Zhao H., Han L., Tong Y., Tan S., Yang K. (2020). Gated Fully Fusion for Semantic Segmentation. AAAI Conf. Artif. Intell..

[40] Li X., You A., Zhu Z., Zhao H., Yang M., Yang K., Tong Y. Semantic Flow for Fast and Accurate Scene Parsing. Proceedings of the European Conference on Computer Vision (ECCV).

[41] Vaswani A., Shazeer N., Parmar N., Uszkoreit J., Jones L., Gomez A.N., Kaiser L.U., Polosukhin I. Attention is All you Need. Proceedings of the Advances in Neural Information Processing Systems.

[42] Wang X., Girshick R., Gupta A., He K. Non-local Neural Networks. Proceedings of the IEEE Conference on Computer Vision and Pattern Recognition (CVPR).

[43] Fu J., Liu J., Tian H., Li Y., Bao Y., Fang Z., Lu H. Dual Attention Network for Scene Segmentation. Proceedings of the IEEE Conference on Computer Vision and Pattern Recognition (CVPR).

[44] Zhang H., Zhang H., Wang C., Xie J. Co-Occurrent Features in Semantic Segmentation. Proceedings of the IEEE Conference on Computer Vision and Pattern Recognition (CVPR).

[45] Yuan Y., Wang J. (2018). OCNet: Object Context Network for Scene Parsing. arXiv.

[46] Yuan Y., Chen X., Wang J. Object-Contextual Representations for Semantic Segmentation. Proceedings of the European Conference on Computer Vision (ECCV).

[47] Araslanov N., Roth S. Single-Stage Semantic Segmentation From Image Labels. Proceedings of the IEEE Conference on Computer Vision and Pattern Recognition (CVPR).

[48] Huang Z., Wang X., Wang J., Liu W., Wang J. Weakly-Supervised Semantic Segmentation Network with Deep Seeded Region Growing. Proceedings of the IEEE Conference on Computer Vision and Pattern Recognition (CVPR).

[49] Lee J., Kim E., Lee S., Lee J., Yoon S. FickleNet: Weakly and Semi-Supervised Semantic Image Segmentation Using Stochastic Inference. Proceedings of the IEEE Conference on Computer Vision and Pattern Recognition (CVPR).

[50] Papandreou G., Chen L.C., Murphy K.P., Yuille A.L. Weakly-and Semi-Supervised Learning of a Deep Convolutional Network for Semantic Image Segmentation. Proceedings of the IEEE International Conference on Computer Vision (ICCV).

[51] Dai J., He K., Sun J. BoxSup: Exploiting Bounding Boxes to Supervise Convolutional Networks for Semantic Segmentation. Proceedings of the IEEE International Conference on Computer Vision (ICCV).

[52] Khoreva A., Benenson R., Hosang J., Hein M., Schiele B. Simple Does It: Weakly Supervised Instance and Semantic Segmentation. Proceedings of the IEEE Conference on Computer Vision and Pattern Recognition (CVPR).

[53] Song C., Huang Y., Ouyang W., Wang L. Box-Driven Class-Wise Region Masking and Filling Rate Guided Loss for Weakly Supervised Semantic Segmentation. Proceedings of the IEEE Conference on Computer Vision and Pattern Recognition (CVPR).

[54] Chen L., Lopes R.G., Cheng B., Collins M.D., Cubuk E.D., Zoph B., Adam H., Shlens J. Leveraging Semi-Supervised Learning in Video Sequences for Urban Scene Segmentation. Proceedings of the European Conference on Computer Vision (ECCV).

[55] Feng Z., Zhou Q., Gu Q., Tan X., Cheng G., Lu X., Shi J., Ma L. (2020). DMT: Dynamic Mutual Training for Semi-Supervised Learning. arXiv.

[56] Olsson V., Tranheden W., Pinto J., Svensson L. ClassMix: Segmentation-Based Data Augmentation for Semi-Supervised Learning. Proceedings of the IEEE Winter Conference on Applications of Computer Vision (WACV).

[57] Mittal S., Tatarchenko M., Brox T. (2021). Semi-Supervised Semantic Segmentation With High- and Low-Level Consistency. IEEE Trans. Pattern Anal. Mach. Intell..

[58] Souly N., Spampinato C., Shah M. Semi Supervised Semantic Segmentation Using Generative Adversarial Network. Proceedings of the IEEE International Conference on Computer Vision (ICCV).

[59] Zou Y., Zhang Z., Zhang H., Li C.L., Bian X., Huang J.B., Pfister T. PseudoSeg: Designing Pseudo Labels for Semantic Segmentation. Proceedings of the International Conference on Learning Representations (ICLR).

[60] Chen X., Yuan Y., Zeng G., Wang J. Semi-Supervised Semantic Segmentation with Cross Pseudo Supervision. Proceedings of the IEEE Conference on Computer Vision and Pattern Recognition (CVPR).

[61] Zhao X., Vemulapalli R., Mansfield P.A., Gong B., Green B., Shapira L., Wu Y. Contrastive Learning for Label Efficient Semantic Segmentation. Proceedings of the IEEE International Conference on Computer O Vision (ICCV).

[62] Khosla P., Teterwak P., Wang C., Sarna A., Tian Y., Isola P., Maschinot A., Liu C., Krishnan D. Supervised Contrastive Learning. Proceedings of the Advances in Neural Information Processing Systems.

[63] Hernández-García A., König P. (2018). Data augmentation instead of explicit regularization. arXiv.

[64] Zou Y., Yu Z., Liu X., Kumar B.V.K.V., Wang J. Confidence Regularized Self-Training. Proceedings of the IEEE International Conference on Computer Vision (ICCV).

[65] Saito K., Kim D., Sclaroff S., Darrell T., Saenko K. Semi-Supervised Domain Adaptation via Minimax Entropy. Proceedings of the IEEE International Conference on Computer Vision (ICCV).

[66] Yu L., Liu X., van de Weijer J. (2020). Self-Training for Class-Incremental Semantic Segmentation. arXiv.

[67] Xu T.B., Liu C.L. (2019). Data-Distortion Guided Self-Distillation for Deep Neural Networks. AAAI Conf. Artif. Intell..

[68] Wang X., Hua Y., Kodirov E., Clifton D.A., Robertson N.M. ProSelfLC: Progressive Self Label Correction for Training Robust Deep Neural Networks. Proceedings of the IEEE Conference on Computer Vision and Pattern Recognition (CVPR).

[69] Li J., Wong Y., Zhao Q., Kankanhalli M.S. Learning to Learn From Noisy Labeled Data. Proceedings of the IEEE Conference on Computer Vision and Pattern Recognition (CVPR).

[70] Liu Y., Shu C., Wang J., Shen C. (2020). Structured Knowledge Distillation for Dense Prediction. IEEE Trans. Pattern Anal. Mach. Intell..

[71] Wang Y., Zhou W., Jiang T., Bai X., Xu Y. Intra-class Feature Variation Distillation for Semantic Segmentation. Proceedings of the European Conference on Computer Vision (ECCV).

[72] Park S., Heo Y.S. (2020). Knowledge Distillation for Semantic Segmentation Using Channel and Spatial Correlations and Adaptive Cross Entropy. Sensors.

[73] Yuan L., Tay F.E., Li G., Wang T., Feng J. Revisiting Knowledge Distillation via Label Smoothing Regularization. Proceedings of the IEEE Conference on Computer Vision and Pattern Recognition (CVPR).

[74] Zhang L., Song J., Gao A., Chen J., Bao C., Ma K. Be Your Own Teacher: Improve the Performance of Convolutional Neural Networks via Self Distillation. Proceedings of the IEEE International Conference on Computer Vision (ICCV).

[75] Kim K., Ji B., Yoon D., Hwang S. Self-Knowledge Distillation With Progressive Refinement of Targets. Proceedings of the IEEE International Conference on Computer Vision (ICCV).

[76] Chollet F. Xception: Deep learning with depthwise separable convolutions. Proceedings of the IEEE conference on computer vision and pattern recognition (CVPR).

[77] He K., Zhang X., Ren S., Sun J. (2015). Deep Residual Learning for Image Recognition. arXiv.

[78] Deng J., Dong W., Socher R., Li L.J., Li K., Fei-Fei L. Imagenet: A large-scale hierarchical image database. Proceedings of the IEEE Conference on Computer Vision and Pattern Recognition (CVPR).

[79] Guo C., Pleiss G., Sun Y., Weinberger K.Q. On Calibration of Modern Neural Networks. Proceedings of the 34th International Conference on Machine Learning (ICML).

